# Hepatitis C Virus NS5A Inhibits YAP1-HSC70 Interaction, Thereby Preventing YAP1 Degradation Via Chaperone-Mediated Autophagy

**DOI:** 10.24546/0100497746

**Published:** 2025-10-03

**Authors:** MARIA ALETHEA SEPTIANASTITI, CHIEKO MATSUI, ZIHAN XU, FRANSISCA PUSPITASARI, LIN DENG, TAKAYUKI ABE, IKUO SHOJI

**Affiliations:** 1 Division of Infectious Disease Control, Center for Infectious Diseases, Kobe University Graduate School of Medicine, Kobe, Japan; 2 Faculty of Medicine, Public Health and Nursing, Universitas Gadjah Mada, Yogyakarta, Indonesia; 3 Division of Virology, Niigata University Graduate School of Medical and Dental Sciences, Niigata, Japan

**Keywords:** HCV, NS5A, YAP1, HSC70, Chaperone-mediated autophagy

## Abstract

Hepatitis C virus (HCV) infection induces chaperone-mediated autophagy (CMA), where the HCV NS5A protein promotes the binding of the molecular chaperone HSC70 to substrate proteins containing a KFERQ motif. HSC70 recognizes this pentapeptide motif and transports substrates to lysosomes for degradation. In this study, we identified a KFERQ motif (^324^ELLRQ^328^) in the YAP1 protein, a key regulator of the Hippo pathway, and examined its interaction with HSC70 during HCV infection. To determine whether HSC70 directly binds to this motif, we generated four YAP1 mutants with specific alterations in the KFERQ motif and assessed their binding to HSC70. Among these, the R327A/Q328A mutant showed the greatest reduction in HSC70 binding, indicating that the sequence ^324^ELLRQ^328^ functions as the KFERQ motif in YAP1. Further analysis revealed that YAP1 binds to the substrate-binding domain of HSC70. Moreover, shRNA-mediated knockdown of LAMP2A, a critical receptor for CMA, led to increased levels of YAP1, confirming that YAP1 is indeed a CMA substrate. Notably, HSC70 was also found to interact with phosphorylated YAP1 at serine 127 (S127). Although NS5A did not bind directly to YAP1, NS5A expression reduced the binding between YAP1 and HSC70. This suggests that under normal conditions, YAP1 is recognized by HSC70 and degraded via CMA. However, during HCV infection, NS5A interferes with YAP1-HSC70 interaction, preventing YAP1 from being degraded. As a result, YAP1 accumulates in the cytoplasm and subsequently translocates to the nucleus, where YAP1 may activate genes involved in cell proliferation and survival. This mechanism may contribute to HCV-induced pathogenesis.

## INTRODUCTION

Many viruses, including hepatitis C virus (HCV), exploit cellular protein degradation systems to promote viral propagation and pathogenesis. Autophagy is a key cellular process that delivers cytoplasmic components to lysosomes for degradation. While chaperone-mediated autophagy (CMA) was once considered to be the only selective lysosomal autophagy pathway, recent studies have identified endosomal microautophagy (eMI) as another pathway capable of selective substrate degradation [1].

We previously demonstrated that HCV enhances CMA by promoting the interaction between the cellular chaperone HSC70 and the transcription factor HNF-1α via the HCV NS5A protein, leading to lysosomal degradation of HNF-1α and subsequent downregulation of glucose transporter 2 expression [2–5]. Additionally, we found that HCV infection augments eMI by facilitating the interaction between HSC70 and diacylglycerol O-acyltransferase 1 (DGAT1) through NS5A, resulting in the selective degradation of DGAT1 [6]. Despite these findings, the broader mechanisms by which HCV exploit selective lysosomal degradation pathways, such as CMA and eMI, and their implications in viral life cycle and disease progression remain largely unclear.

The substrate selectivity in these pathways governed by the presence of a specific pentapeptide sequence, known as the KFERQ motif, in the target protein [7, 8]. The KFERQ motif typically includes one or two positively charged residues (lysine [K] and arginine [R]); one or two hydrophobic residues (phenylalanine [F], isoleucine [I], leucine [L], and valine [V]); one negatively charged residues (aspartic acid [D] or glutamic acid [E]); and one glutamine (Q) located on either side of the pentapeptide [9–11]. Cuervo and colleagues have developed a freely accessible web tool, KFERQ finder V0.8 (https://rshine.einsteinmed.edu/), to identify such motifs in protein sequences [12].

The interaction between HSC70 and the KFERQ-motif containing proteins is crucial for both CMA and eMI. In CMA, HSC70 recognizes the KFERQ motif and delivers the substrate to LAMP2A on the lysosomal membrane, where the substrate is unfolded and translocated for degradation. On the other hand, eMI involves the sequestration of substrate proteins within the late endosomal membrane. Following substrate binding, HSC70 interacts with phosphatidylserine (PS) on the endosomal membrane, triggering membrane invagination. The process is mediated by tumor susceptibility gene (TSG) 101, a component of endosomal sorting complex required for transport (ESCRT I), along with vacuolar protein sorting-associated protein (VPS) 4A, VPS4B, and Alix [13–15].

HSC70 is a 70-kDa ATP-dependent molecular chaperone that plays a central role in various cellular processes, including the prevention of protein misfolding and aggregation under both normal and stressed conditions [16]. Structurally, HSC70 consists of a nucleotide-binding domain (NBD) and a substrate-binding domain (SBD) [17]. The SBD is further subdivided into a β-sandwich subdomain containing a hydrophobic cleft for substrate binding [18], a helical LID subdomain that regulates access to the cleft and a disordered C-terminal domain (CTD), which serves as a site of post-translational modification and protein-protein interaction [19, 20].

The transcription factor Yes-associated protein 1 (YAP1) is a key effector of Hippo pathway and plays a critical role in liver pathogenesis by promoting cell proliferation, cell survival, migration, and metastasis [21–23]. When the Hippo pathway is active, YAP1 is phosphorylated at serine 127, enabling its interaction with 14-3-3 proteins and resulting in cytoplasmic retention. In contrast, when the Hippo pathway is inactivated, unphosphorylated YAP1 translocates to the nucleus, where it activates transcription of genes involved in cell proliferation. Recent studies have shown that YAP1 is a CMA substrate in HuS, Hep3B, and HepG2 cells. Impaired degradation of YAP1 via CMA has been linked to increased proliferation and migration in both normal and hepatocellular carcinoma cells [24].

We previously demonstrated that HCV NS5A facilitates CMA and eMI by enhancing HSC70-substrate interactions. In this study, we identified a KFERQ motif within the YAP1 protein sequence, suggesting that YAP1 is a potential substrate for CMA-mediated degradation. Thus, we investigated whether YAP1 is targeted for degradation via HCV-induced CMA or eMI. Our results showed that NS5A did not directly bind YAP1. Instead, NS5A disrupts the interaction between HSC70 and YAP1, thereby preventing YAP1 degradation via CMA. Since increased YAP1 activity is linked to increased cell proliferation and migration, the ability of HCV to protect YAP1 from CMA-mediated degradation may represent a novel mechanism underlying HCV-induced pathogenesis.

## MATERIALS AND METHODS

### Cell culture and viruses

The human hepatoma cell line, Huh-7.5 cells, was provided by Dr. Charles M. Rice (The Rockefeller University, New York, NY) [25]. Cells were maintained in high-glucose Dulbecco’s modified Eagle’s medium (DMEM) supplemented with L-glutamine and phenol red (044-29765; Fuji Film Wako Pure Chemical Industries, Osaka, Japan), 50 IU/ml penicillin, 50 μg/ml streptomycin (15-140-122; Gibco, Grand Island, NY), 10% heat-inactivated fetal bovine serum (S1760-500; Biowest, Nuaillé, France), and 0.1 mM non-essential amino acids (Invitrogen, Carlsbad, CA). Cells were incubated at 37°C in a 5% CO_2_ incubator. Phosphate-buffered saline (PBS) without calcium and magnesium (PBS [–]; 05913; Nissui Pharmaceutical Co., Ltd., Tokyo, Japan) was used for washing. Transfections were carried out using FuGENE 6 transfection reagents (E269A; Promega, Madison, WI). The pFL-J6/JFH1 plasmid encoding the entire viral genome of a chimeric strain of HCV-2a, J6/JFH1 was kindly provided by Dr. C.M. Rice [26]. The HCV genome RNA was synthesized in vitro using pFL-J6/JFH1 as a template and was transfected into Huh-7.5 cells by electroporation [27–29]. The virus produced in the culture supernatant was used for infection experiments [28]. Huh-7 cells stably harboring the HCV-1b full-genome replicon (FGR) derived from Con1 (RCYM1) were also utilized [30].

### Expression plasmids

Expression plasmids encoding FLAG-tagged or Myc-His_6_-tagged NS5A and its deletion mutants have been previously described [2–4, 6, 31, 32]. The plasmid expressing HSC70 was also described previously [2, 6]. To clone YAP1, total RNA was extracted from Huh-7.5 cells and reverse-transcribed, followed by PCR amplification using the following primers:

- Forward: 5′-TCGAGCTCAGCGGCCGCCATGGATCCCGGGCAGCAG-3′
- Reverse: 5′-AGTGAATTCGCGGCCGCTTATAACCATGTAAGAAA-3′

The amplified PCR product was purified and inserted into NotI site of pCAG-HA vector using the In-Fusion HD cloning kit (Takara Bio USA, Inc., 639649). Point mutations in YAP1 were introduced by overlap extension PCR using pCAG-HA-YAP1 as a template. Primer sequences used to generate the mutants were as follows:

Q323A:

- Forward: 5′-CGGCTGAAACAGGCAGAACTGCTTCGGCAG-3′
- Reverse: 5′-CTGCCGAAGCAGTTCTGCCTGTTTCAGCCG-3′

Q323A/E324A

- Forward: 5′-CGGCTGAAACAGGCAGCACTGCTTCGGCAG-3′
- Reverse: 5′-CTGCCGAAGCAGTGCTGCCTGTTTCAGCCG-3′

R327A/Q328A

- Forward: 5′-CAAGAACTGCTTGCGGCGGCAATGCGGAAT-3′
- Reverse: 5′-ATTCCGCATTGCCGCCGCAAGCAGTTCTTG-3′

R327Q/Q328R

- Forward: 5′-CAAGAACTGCTTCAGCGGGCAATGCGGAAT-3′
- Reverse: 5′-ATTCCGCATTGCCCGCTGAAGCAGTTCTTG-3′

All constructs were confirmed by DNA sequencing (Eurofins Genomics).

### Antibodies and reagents

The mouse monoclonal antibodies (mAbs) used in this study were anti-FLAG (M2) mAb (F-3165; Sigma-Aldrich, St. Louis, MO, USA), anti-HSC70 (B-6) mAb (sc-7298; Santa Cruz Biotechnology, Dallas, TX, USA), anti-c-Myc (9E10) mAb (sc-40; Santa Cruz Biotechnology), anti-ß-actin mAb (A-5441; Sigma-Aldrich), anti-DDDDK-tag mAb (M185-3L; MBL International Corporation, Woburn, MA, USA), anti-YAP1 (63.7) mAb (sc-101199; Santa Cruz Biotechnology), and anti-VPS4B (A-11) mAb (sc-377162; Santa Cruz Biotechnology). The rabbit polyclonal antibodies (pAbs) used in this study were anti-HA pAb (H-6908; Sigma-Aldrich), anti-YAP1 mAb (4912S; Cell Signaling Technology, Danvers, MA, USA), anti-Phospho-YAP1 (Ser127) mAb (4911S; Cell Signaling Technology), anti-LAMP2A pAb (ab18528; Abcam, Cambridge, UK), anti-DDDDK-tag pAb (PM020; MBL International Corporation) and anti-NS5A (2914-1) pAb (a kind gift from T. Wakita, National Institute of Infectious Diseases, Tokyo). Horseradish peroxidase (HRP)-conjugated anti-mouse IgG (7076S; Cell Signaling Technology) and HRP-conjugated anti-rabbit IgG (7074S; Cell Signaling Technology) were used as secondary antibodies.

### Immunoblot analysis

Immunoblot analysis was performed as described previously [33, 34]. Cell lysates were resolved on 10% or 15% sodium dodecyl sulfate-polyacrylamide gel electrophoresis (SDS-PAGE) and transferred to 0.45 μm polyvinylidene difluoride membrane (PVDF) (Immobilon-P; Millipore, Billerica, MA). Membranes were probed with primary antibodies, followed by horseradish peroxidase (HPR)-conjugated secondary antibody. The positive bands were visualized using Amersham enhanced chemiluminescence (ECL) western blotting detection reagents (RPN2106; Cytiva, Sigma-Aldrich). Band intensities were quantified using ImageJ software (version 1.53r).

### Immunoprecipitation

Cells were lysed in buffer containing 150 mM NaCl, 50 mM Tris-HCl (pH 7.5), 1% NP-40, 0.1% SDS, 1% sodium deoxycholic acid, 1 mM EDTA, and protease inhibitor cocktail (cOmplete™, EDTA-free, Roche Molecular Biochemicals, Mannheim, Germany). The lysate was centrifuged at 12,500 × g for 15 min at 4°C, and the supernatants were subjected to immunoprecipitation using appropriate antibodies. Immunoprecipitation was performed as described [35, 36]. Briefly, the cell lysates were immunoprecipitated with anti-FLAG M2 affinity gel (Sigma-Aldrich Co., A2220) or Protein A-Sepharose 4 Fast Flow (GE17-5280-04; GE Healthcare, Buckinghamshire, UK) pre-incubated with appropriate antibodies. Samples were rotated at 4°C for 4 h or overnight. After five washes with the lysis buffer, the immunoprecipitated samples were analyzed by immunoblotting.

### RNA interference and generation of stable knockdown cells

The target sequences for the short hairpin RNAs (shRNAs) against LAMP2A, VPS4B, and scramble control were as follows:

- shLAMP2A: 5′-GGCAGGAGUACUUAUUCUA-3′
- shVPS4B: 5′-AGCGAUAGAUCUGGCUAGCAA-3′
- shScramble: 5′-GGACAUCGACGGCUUUAUA-3′

A pair of complementary oligonucleotides encoding the shRNA targeting VPS4B was synthesized, annealed, and inserted into the pSilencer 2.1-U6 puro vector according to the manufacturer’s instructions (AM5762; Ambion, Austin, TX, USA). A pair of complementary oligonucleotides encoding the shRNA targeting LAMP2A was synthesized, annealed, and inserted into the pSilencer 2.1-U6 hygro vector (AM5760; Ambion). A pair of complementary oligonucleotides encoding the shRNA targeting scrambled control was inserted into the pSilencer 2.1-U6 hygro vector similarly. Huh-7.5 cells were transfected with these plasmids, and stable knockdown cell lines were established by selecting drug-resistant clones using 1 μg/ml puromycin (P9620; Sigma-Aldrich) or 200 μg/ml hygromycin B (09287-84; Nacalai Tesque, Kyoto, Japan) depending on the resistance cassette in the vector (6, 37).

### Immunofluorescence staining

Huh-7.5 cells cultured on glass coverslips were fixed with 4% paraformaldehyde at room temperature (RT) for 15 min. After being washed with PBS, the cells were permeabilized with PBS containing 0.1% Triton X-100 for 15 min at RT. To block nonspecific binding, the cells were incubated in PBS supplemented with 1% bovine serum albumin (BSA) (01859-47; Nacalai Tesque) for 60 min. Subsequently, the cells were incubated for 60 min at RT with a mouse monoclonal anti-YAP1 antibody and a rabbit polyclonal anti-NS5A antibody, both diluted in 1% BSA in PBS. Afterwards, the cells were washed three times with PBS and further incubated for 60 min at RT with Alexa Fluor™ 594-conjugated anti-mouse IgG (A11005; Invitrogen) and Alexa Fluor™ 488-conjugated anti-rabbit IgG (A11008; Invitrogen) diluted in 1% BSA in PBS. Finally, the cells were washed four times with PBS, mounted on glass slides, and imaged using a confocal microscope (LSM 700, Carl Zeiss, Oberkochen, Germany).

### Cell fractionation assay

Cytoplasmic and nuclear fractions were prepared from HCV-infected and mock-infected Huh-7.5 cells using NE-PER™ Nuclear and Cytoplasmic Extraction Reagents (78835; Thermo Fisher Scientific) according to the manufacturer’s instructions. The resulting lysates were subjected to immunoblot analysis.

## RESULTS

### The KFERQ motif at amino acid (aa) positions 324–328 of YAP1 is essential for interaction with HSC70

**Fig. 1:**
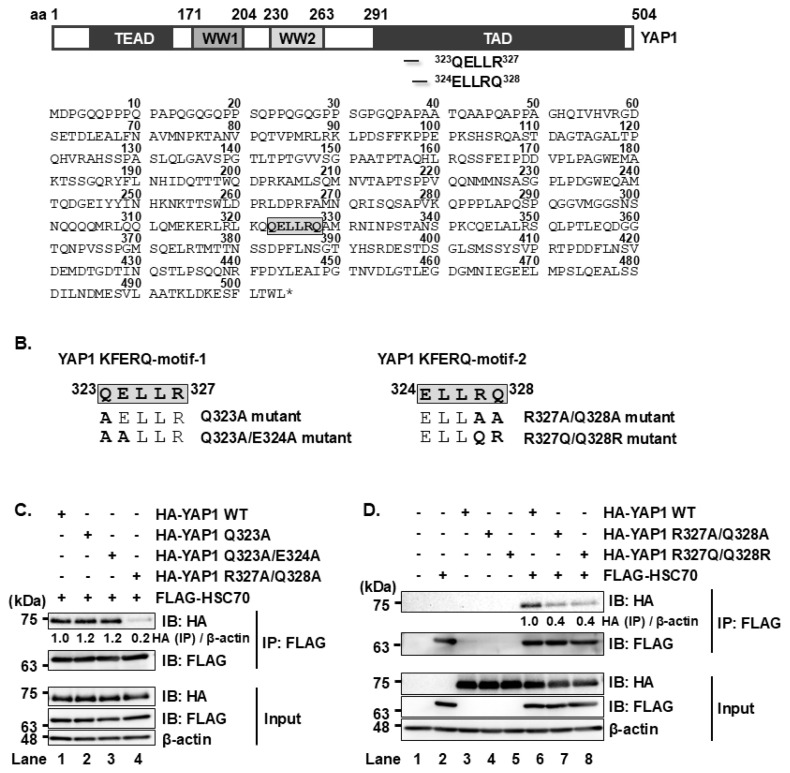
HSC70 recognizes and binds to the KFERQ motif ^324^ELLRQ^328^ of YAP1 **(A)** Schematic representation of the YAP1 protein and its amino acid sequences. The KFERQ-like motif is located within the region spanning amino acids 323–327 and 324–328 of YAP1. **(B)** Expression plasmids were constructed for YAP1 mutants with disruptions in the KFERQ motif: HA-YAP1 Q323A and Q323A/E324A (motif-1), and R327A/Q328A and R327Q/Q328R (motif-2). **(C, D)** Huh-7.5 cells were seeded and cultured for 12 h, followed by transfection with pCAG-HA-YAP1 wild-type (WT) or the indicated KFERQ motif mutants along with the plasmid expressing FLAG-HSC70. At 48 h post-transfection, cells were harvested and lysed. Lysates were subjected to immunoprecipitation using anti-FLAG mouse mAb. Both input and immunoprecipitated samples were analyzed by immunoblotting using anti-FLAG mouse mAb and anti-HA rabbit pAb. Band intensities were quantified by densitometric analysis using ImageJ. Relative protein levels were normalized to β-actin and are presented below the corresponding lanes. Data shown are representative of at least three independent experiments.

To identify potential KFERQ motifs in YAP1, we analyzed its aa sequence according to the consensus sequence rules for KFERQ motifs. This analysis revealed two possible motifs: one spanning aa residues 323–327 (QELLR), and another spanning aa residues 324–328 (ELLRQ) (Figs. 1A and 1B). Both sequences conform to the KFERQ consensus.

To assess the functional relevance of these motifs in mediating the interaction between YAP1 and HSC70, we generated point mutants of YAP1 expression plasmids: pCAG-HA-YAP1 Q323A, pCAG-HA-YAP1 Q323A/E324A, pCAG-HA-YAP1 R327A/Q328A, and pCAG-HA-YAP1 R327Q/Q328R (Fig. 1B). Co-immunoprecipitation analyses revealed that FLAG-HSC70 was co-precipitated with wild-type (WT) HA-YAP1, as well as with HA-YAP1 Q323A and HA-YAP1 Q323A/E324A (Fig. 1C, first panel, lanes 1, 2, and 3). In contrast, HA-YAP1 R327A/Q328A and HA-YAP1 R327Q/Q328R exhibited significantly reduced binding to FLAG-HSC70 (Fig. 1C, first panel, lane 4; Fig. 1D, first panel, lanes 7 and 8). These results indicate that the KFERQ motif at aa 324–328 (ELLRQ) is crucial for the YAP1-HSC70 interaction.

### The substrate-binding domain of HSC70 mediates interaction with YAP1

**Fig 2:**
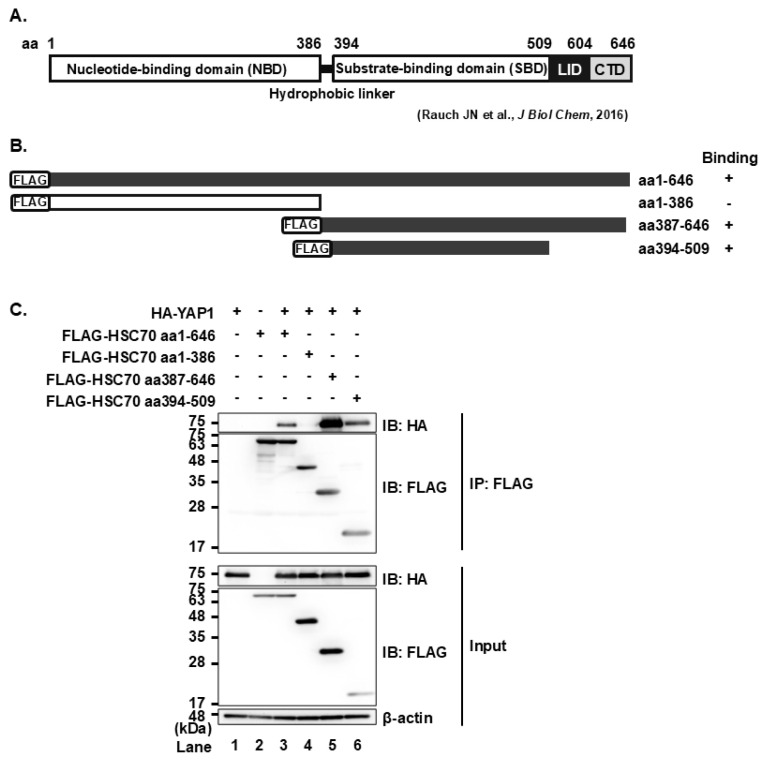
Mapping of the YAP1-binding site on the chaperone protein HSC70 **(A)** Schematic representation of the HSC70 protein. HSC70 consists of two major domains: the nucleotide-binding domain (aa 1–386) and the substrate-binding domain (aa 394–646). The substrate-binding domain is further divided into a 10-kDa helical LID domain (aa 509–604) and a 5-kDa intrinsically disordered C-terminal domain (CTD: aa 605–646). **(B)** Each HSC70 deletion mutant contains an N-terminal FLAG-tag, represented by the rectangular region. Filled boxes indicate proteins that bind YAP1, while an open box indicates the protein that does not. **(C)** Huh-7.5 cells were seeded and cultured for 12 h, then transfected with pCAG-HA-YAP1 and each of the FLAG-tagged HSC70 mutant plasmid as indicated. At 48 h post-transfection, cells were harvested, and lysates were immunoprecipitated with anti-FLAG mouse mAb. Input and immunoprecipitated samples were analyzed by immunoblotting with anti-FLAG mouse mAb or anti-HA rabbit pAb. Data shown are representative of three independent experiments.

To map the region of HSC70 responsible for binding to YAP1, we performed co-immunoprecipitation analysis using a series of FLAG-tagged HSC70 deletion mutants (Fig. 2B). HSC70 comprises two main domains: the N-terminal nucleotide-binding domain (NBD, aa 1–386), and the C-terminal substrate-binding domain (SBD, aa 394–646). The SBD is further divided into a 10-kDa helical LID domain (aa 509–604) and a 5-kDa dynamically unstructured C-terminal domain (CTD, aa 605–646). The HA-YAP1 proteins were coimmunoprecipitated with all of the FLAG-HSC70 proteins except FLAG-HSC70 (aa 1–386) (Fig. 2C, upper panel, lanes 3–6) with the use of anti-FLAG mAb. These results suggest that the SBD domain on HSC70 amino acid consisting of aa 394 to 509 is important for YAP1 binding. The SBD domain is critical for binding to the substrate protein for degradation. This result suggests that YAP1 is recognized by HSC70 as a substrate for selective degradation.

### YAP1 is degraded via chaperone-mediated autophagy

**Fig. 3:**
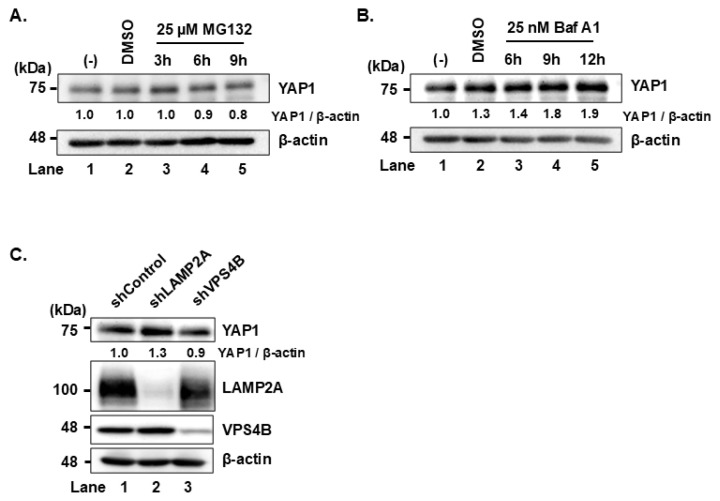
YAP1 is degraded via chaperone-mediated autophagy in Huh-7.5 cells **(A)** Huh-7.5 cells were treated with 25μM MG132 for 3, 6, and 9 h. Cell lysates were collected and analyzed by immunoblotting with the indicated antibody. β-actin served as a loading control. The relative levels of the proteins were quantified by densitometric analysis using ImageJ and are indicated below the respective lanes. **(B)** Huh-7.5 cells were plated and cultured for 12 h. Cells were treated with 25nM Bafilomycin A1 (Baf A1) for 6, 9, and 12 h before harvested and analyzed by immunoblotting with indicated antibody. The level of β-actin served as loading control. The relative levels of the proteins were quantified by densitometric analysis using ImageJ and are indicated below the respective lanes. **(C)** shControl, shLAMP2A-Huh-7.5 cells, and shVPS4B-Huh-7.5 cells were cultured for 72 h prior to harvesting and analyzed using immunoblotting with the indicated antibodies. β-actin served as a loading control. The relative levels of the proteins were quantified by densitometric analysis using ImageJ and are indicated below the respective lanes. Data shown are representative of three independent experiments.

To further investigate whether YAP1 is degraded via lysosomal or proteasomal degradation pathway, we treated the Huh-7.5 cells with the lysosomal inhibitor bafilomycin A1 or the proteasome inhibitor MG132. YAP1 expression increased upon bafilomycin A1 treatment but remained unchanged with MG132, suggesting lysosomal, rather than proteasomal, degradation (Figs. 3A and 3B, first panel). LAMP2A serves as a specific receptor for the CMA pathway, whereas VPS4B is essential for eMI. Therefore, to determine whether YAP1 is degraded via CMA or eMI, we generated stable LAMP2A knockdown Huh-7.5 cells (shLAMP2A-Huh-7.5 cells) and stable VPS4B knockdown Huh-7.5 cells (shVPS4B-Huh-7.5 cells) using short hairpin RNA (shRNA). Immunoblot analysis showed an increase in YAP1 levels in shLAMP2A-Huh-7.5 cells than in the shVPS4B-Huh-7.5 cells (Fig 3C, first panel). This finding suggests that YAP1 is degraded via CMA in Huh-7.5 cells.

### HCV NS5A prevents the interaction between HSC70 and YAP1 protein

**Fig. 4:**
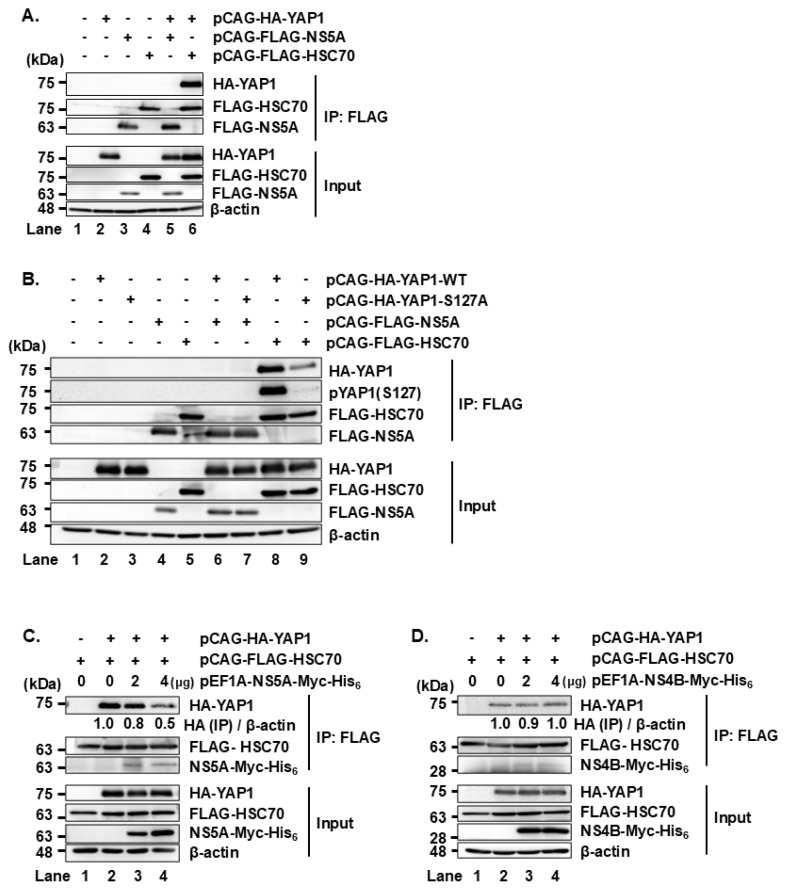
NS5A inhibits the interaction between HSC70 and YAP1 **(A)** Huh-7.5 cells were seeded and cultured for 12 h, followed by transfection with pCAG-HA-YAP1 and either pCAG-FLAG-NS5A or pCAG-FLAG-HSC70. At 48 h post-transfection, cells were harvested, and lysates were immunoprecipitated with anti-FLAG mouse mAb. Input and immunoprecipitated samples were analyzed by immunoblotting with the indicated antibodies. **(B)** Huh-7.5 cells were transfected with pCAG-HA-YAP1 WT or S127A mutant, together with pCAG-FLAG-NS5A or pCAG-FLAG-HSC70. At 48 h post-transfection, cells were harvested, and lysates were immunoprecipitated with anti-FLAG mouse mAb. Input and immunoprecipitated samples were analyzed by immunoblotting with the indicated antibodies. **(C, D)** Huh-7.5 cells were transfected with increasing amounts of either pEF1A-NS5A-Myc-His_6_ or pEF1A-NS4B-Myc-His_6_. At 48 h post-transfection, cells were harvested and lysed. Lysates were subjected to immunoprecipitation using anti-FLAG mAb. Both input and immunoprecipitated samples were analyzed by immunoblotting using anti-FLAG mouse mAb, anti-HA rabbit pAb, and anti-c-Myc mouse mAb. Band intensities were quantified by densitometric analysis using ImageJ. Relative protein levels were normalized to β-actin and are presented below the corresponding lanes. Data shown are representative of three independent experiments.

To determine whether HCV NS5A protein interacts with YAP1 protein in Huh-7.5 cells, we co-transfected pCAG-HA-YAP1 together with pCAG-FLAG-NS5A. Immunoprecipitation analysis using anti-FLAG mAb revealed that FLAG-HSC70 was co-precipitated with HA-YAP1 protein (Fig. 4A, first panel, lane 6), which is consistent with the results from Fig. 1C and Fig. 1D. However, FLAG-NS5A was not co-precipitated with HA-YAP1 (Fig. 4A, first panel, lane 5). These results suggest that HSC70 interacts with YAP1, whereas NS5A does not bind directly to YAP1 in Huh-7.5 cells.

Furthermore, YAP1 WT was co-precipitated with HSC70, but YAP1 S127A mutant co-precipitation was markedly reduced (Fig. 4B, first panel, lanes 8 and 9). Serine 127 phosphorylation is known to facilitate YAP1 retention in the cytoplasm. Therefore, lack of phosphorylation at this site correlates with increased YAP1 translocation into the nucleus. This result suggests that HSC70 primarily associates with cytoplasmic YAP1.

To investigate a possible role of NS5A in the interaction between HSC70 and YAP1, Huh-7.5 cells were transfected with increasing amounts of either pEF1A-NS5A-Myc-His_6_ or pEF1A-NS4B-Myc-His_6_. Overexpression of NS5A-Myc-His_6_ (Fig. 4C, upper panels, lane 4), but not NS4B-Myc-His_6_ (Fig. 4D, upper panels, lane 4), resulted in significantly reduced co-immunoprecipitation of HA-YAP1 with FLAG-HSC70. These findings suggest that NS5A disrupts interaction between HSC70 and YAP1.

### HCV infection increases YAP1 protein levels via NS5A

**Fig. 5:**
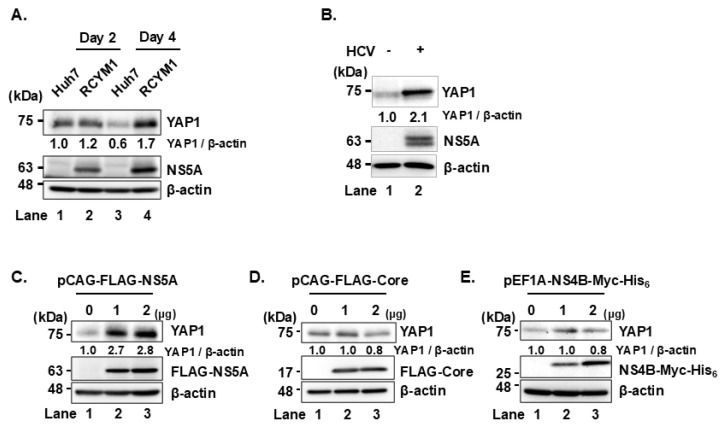
HCV infection increases YAP1 protein levels via NS5A **(A)** HCV genotype 1b replicon cells (RCYM1) and Huh-7 cells as negative controls were plated and cultured, then harvested on days 2 and 4. Cell lysates were prepared and subjected to immunoblot analysis using the indicated antibodies. The level of β-actin served as loading controls. Relative protein expression levels were quantitated by densitometric analysis using ImageJ. **(B)** Huh-7.5 cells were infected with HCV J6/JFH1 at a multiplicity of infection (MOI) of 2. Cells were cultured and harvested at 6 days post infection (dpi). Immunoblot analysis was performed using indicated antibodies. β-actin levels were used as loading controls. Relative protein levels were quantitated by densitometric analysis using ImageJ and are shown below the respective lanes. **(C, D, E)** Huh-7.5 cells were plated and cultured for 12 h. Cells were transfected with pCAG-FLAG-NS5A, pCAG-FLAG-Core, or pEF1A-NS4B-Myc-His_6_. At 48 h post-transfection, cells were harvested and analyzed by immunoblotting with the indicated antibodies. The level of β-actin served as loading controls. Band intensities were quantified by densitometric analysis using ImageJ. Relative protein levels were normalized to β-actin and are presented below the corresponding lanes. Data shown are representative of three independent experiments.

To investigate the effect of HCV infection on YAP1 protein levels, we used Huh-7 cells stably harboring the HCV-1b full genome replicon (RCYM1). Immunoblot analysis revealed that YAP1 protein levels were elevated in HCV-infected cells (RCYM1) compared to control Huh-7 cells (Fig. 5A, first panel, lanes 2 and 4). Consistent with this, increased YAP1 levels were also observed in Huh-7.5 cells infected with HCV J6/JFH1 (Fig. 5B, first panel, lane 2).

To examine whether NS5A contributes to the HCV-induced YAP1 stabilization, Huh-7.5 cells were transfected with increasing amounts of either pCAG-FLAG-NS5A, pCAG-FLAG-Core, or pEF1A-NS4B-Myc-His_6_. NS5A overexpression, but not core or NS4B protein, increased YAP1 protein levels (Fig. 5C, first panel, lanes 2 and 3). These findings indicate that HCV infection promotes YAP1 protein stabilization.

### HCV infection induces YAP1 nuclear translocation

**Fig. 6:**
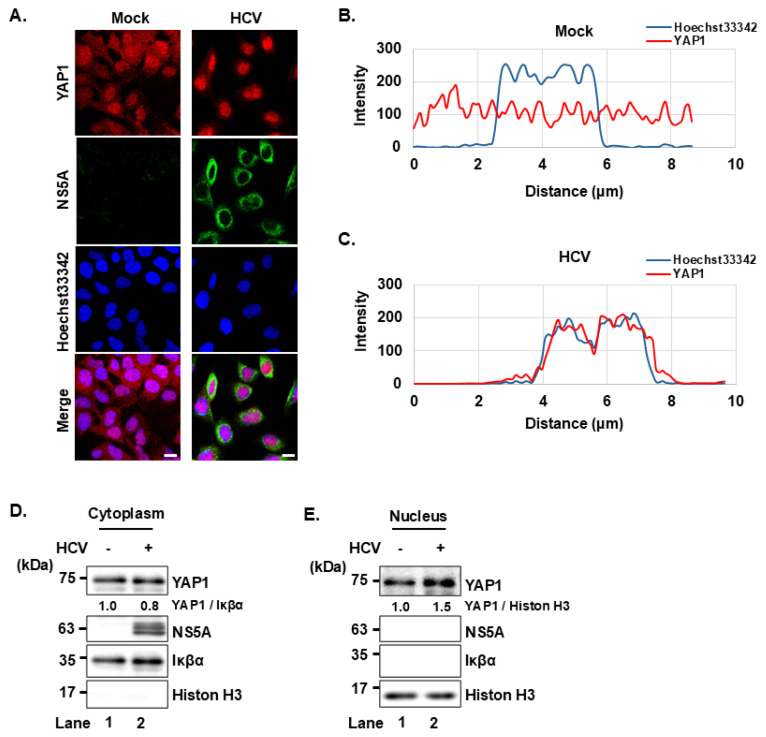
HCV infection induces YAP1 nuclear translocation **(A)** Huh-7.5 cells were plated and cultured for 12 h and infected with HCV J6/JFH1 at multiplicity of infection (MOI) of 2. At 3 days after infection, cells were stained with anti-YAP1 mouse mAb followed by Alexa Fluor 594-conjugated goat anti-mouse IgG (red) and anti-NS5A rabbit pAb followed by Alexa Fluor 488-conjugated goat anti-rabbit IgG (green). Nuclei were counterstained with Hoechst 33342 (blue). Images were acquired using scanning laser confocal microscopy and processed with ImageJ software. Scale bar: 10μm. **(B, C)** A straight line was drawn from the cytoplasm through the nucleus of a representative cell in confocal immunofluorescence images from the mock (B) and HCV-infected (C) groups using ImageJ. The corresponding line scan profiles display the fluorescence intensity (gray value, arbitrary units) of YAP1 (red line) and Hoechst 33342 (blue line) along the measured distance. The degree of overlap between the YAP1 and Hoechst 33342 signals indicates the extent of YAP1 nuclear localization. **(D, E)** Huh-7.5 cells were infected with HCVJ6/JFH1 at an MOI of 2. At 3 days after infection, the cells were harvested and subjected for cell fractionation analysis. Iκβα served as a loading control for the cytoplasmic fraction and Histone H3 served as a loading control for the nuclear fraction. Band intensities were quantified by densitometric analysis using ImageJ. Relative protein levels were normalized to corresponding loading control and are presented below the corresponding lanes. Data shown are representative of three independent experiments.

To further investigate the effect of HCV on YAP1, we performed immunofluorescence staining to examine YAP1 subcellular localization in mock and HCV-infected Huh-7.5 cells. In the mock-infected group, YAP1 was distributed in both the nucleus and cytoplasm, as shown in the fluorescence image and the corresponding line scan profile (Figs. 6A and 6B). In contrast, YAP1 was predominantly localized in the nucleus in HCV-infected cells (Fig. 6A). This is supported by the line scan profile, in which the YAP1 fluorescence intensity peak overlaps with the Hoechst33342 nuclear signal peak, indicating YAP1 accumulation within the nucleus (Fig. 6C).

To biochemically confirm these findings, we performed cell fractionation assay. Consistent with the immunofluorescence staining, the cell fractionation assay showed that HCV infection led to a decrease in cytoplasmic YAP1 (Fig. 6B) and an increase in nuclear YAP1 (Fig. 6C). These findings indicate that HCV infection promotes YAP1 nuclear translocation.

## DISCUSSION

We previously demonstrated that the HCV NS5A protein interacts with the cellular chaperone protein HSC70 during HCV infection. The interaction between NS5A and HSC70 promotes the binding of HSC70 to the KFERQ motif on target proteins, and induces the lysosomal degradation of proteins, such as HNF-1α degradation via CMA and DGAT1 degradation via eMI. The degradation of these host factors contributes to HCV pathogenesis by altering cellular environment in a way that supports efficient viral replication [2–6]. In this study, we focused on the transcriptional co-activator YAP1 protein that plays a crucial role in cell proliferation. We identified possible KFERQ motifs located at aa 323–327 and 324–328 within YAP1 and examined its interaction with HSC70 by generating YAP1 mutants lacking key residues within the possible motifs. Notably, the R327A/Q328A mutation significantly reduced the binding to HSC70, indicating that the ^324^ELLRQ^328^ sequence functions as a bona fide KFERQ motif recognized by HSC70. HSC70 comprises two major domains: NBD and SBD. Using deletion mutants of HSC70, we demonstrated that the SBD is essential for binding to YAP1. Furthermore, treatment with a lysosomal inhibitor, but not a proteasomal inhibitor, led to stabilization of YAP1. These findings indicate that YAP1 is targeted for degradation via selective lysosomal degradation pathway in HSC70-dependent manner.

We next examined whether this HSC70-mediated degradation of YAP1 occurs through CMA or eMI. Since different receptors are involved in each pathway, we knocked down LAMP2A for CMA and VPS4B for eMI to distinguish two pathways. In LAMP2A knockdown cells, YAP1 levels increased, suggesting that YAP1 is degraded via CMA. In contrast, the knockdown of VPS4B had little effect on YAP1 levels, suggesting that eMI is not involved in degradation of YAP1.

**Fig. 7:**
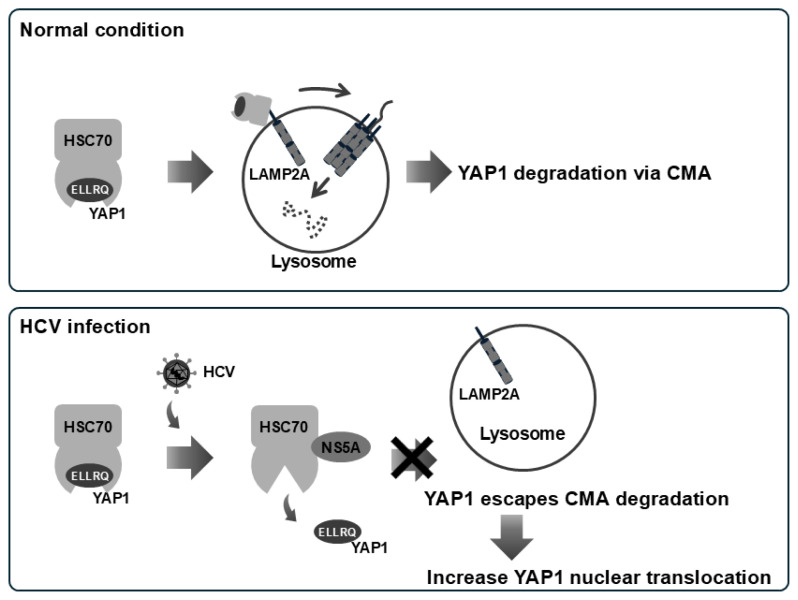
HCV NS5A prevents degradation of YAP1 via chaperon-mediated autophagy Under normal condition, YAP1 is bound to HSC70, leading to YAP1 selective degradation via CMA. Upon HCV infection, however, NS5A disrupts the interaction between YAP1 and HSC70, allowing YAP1 to escape degradation via CMA. As a result, YAP1 translocates into the nucleus. These findings suggest that HCV NS5A contributes to HCV-induced pathogenesis.

To investigate the effect of NS5A on the HSC70–YAP1 interaction, we transfected cells with increasing amounts of NS5A. While NS5A did not directly interact with YAP1, NS5A inhibited HSC70-YAP1 binding in a dose-dependent manner. HCV infection led to increased YAP1 protein levels and enhanced nuclear translocation of YAP1. Importantly, overexpression of NS5A, but not other HCV proteins such as NS4B or Core, led to elevated YAP1 protein levels. We propose a model in which under normal condition, HSC70 interacts with YAP1 to mediate YAP1 degradation via CMA. During HCV infection, NS5A binds to HSC70 and redirects HSC70 to NS5A-binding proteins, thereby preventing HSC70 from targeting YAP1 for degradation. As a result, YAP1 accumulates and translocates into the nucleus (Fig. 7).

We also observed that HA-YAP1 S127A lacking phosphorylation at serine 127 showed reduced binding to HSC70. This suggests that serine 127 phosphorylation is important for YAP1–HSC70 interaction. It is also possible that the reduced HSC70-binding to YAP1 S127A mutant is due to its decreased cytoplasmic localization, as phosphorylation at serine 127 is known to promote cytoplasmic retention of YAP1 [38], where HSC70 can access its targets. Further analysis is required to determine the detailed mechanism.

In addition to our proposed model, in which NS5A directly interacts with HSC70 to disrupt its binding to phosphorylated YAP1, alternative mechanisms may also account for our observations. One possibility is that NS5A modulates upstream components of the Hippo pathway, such as by inhibiting LATS1/2 kinase activity or by activating an unidentified phosphatase, resulting in an increase in non-phosphorylated YAP1. In this study, we demonstrated that HSC70 preferentially binds to YAP1 phosphorylated at serine 127. Therefore, a reduction in YAP1 phosphorylation at this site could weaken its interaction with HSC70, allowing more YAP1 to escape chaperone-mediated autophagy and translocate into the nucleus. While our current findings support a model involving direct NS5A-HSC70 interaction, further studies are required to determine whether NS5A also affects upstream Hippo pathway regulators or phosphatase activity.

Phosphorylation may also broadly influence CMA substrate specificity. For example, certain proteins, such as PLIN2, require phosphorylation to become CMA substrates, whereas others, like phosphoprotein enriched in diabetes (PED), are degraded only in their unphosphorylated state [39–42]. It has been proposed that phosphorylation of serine, threonine, or tyrosine residues near an incomplete KFERQ-like motif can introduce a negative charge, thereby converting it into a functional CMA-targeting signal [10]. However, YAP1 already contains a canonical KFERQ motif, and Ser127 is not located within this motif, suggesting an alternative mechanism–perhaps a phosphorylation-induced conformational change that enhances HSC70 accessibility. Similar regulatory mechanisms have been described for hypoxia-inducible factor 1-alpha (HIF1α), which requires ubiquitination, and mammalian Ste 20-like kinase 1 (MST1), which becomes accessible to CMA only after deacetylation [43, 44].

In conclusion, our findings reveal a novel mechanism by which NS5A disrupts the HSC70-YAP1 interaction, thereby stabilizing YAP1 by inhibiting its CMA-mediated degradation and promoting nuclear translocate of YAP1. Nuclear YAP1 may subsequently activate target genes involved in cell proliferation, potentially creating a favorable environment for viral replication. Further studies are warranted to clarify the mechanism of HCV-induced YAP1 nuclear translocation and to explore its broader implications in viral pathogenesis.
